# Supplementary figures and images for: Exploring HbA1c variation between Australian diabetes centres: The impact of centre-level and patient-level factors
Source: PLoS One. 2022 Feb 4;17(2):e0263511. doi: 10.1371/journal.pone.0263511 (PMC8815864; doi:10.1371/journal.pone.0263511)

***S1.*** *ANDA Data Collection Form 2019*


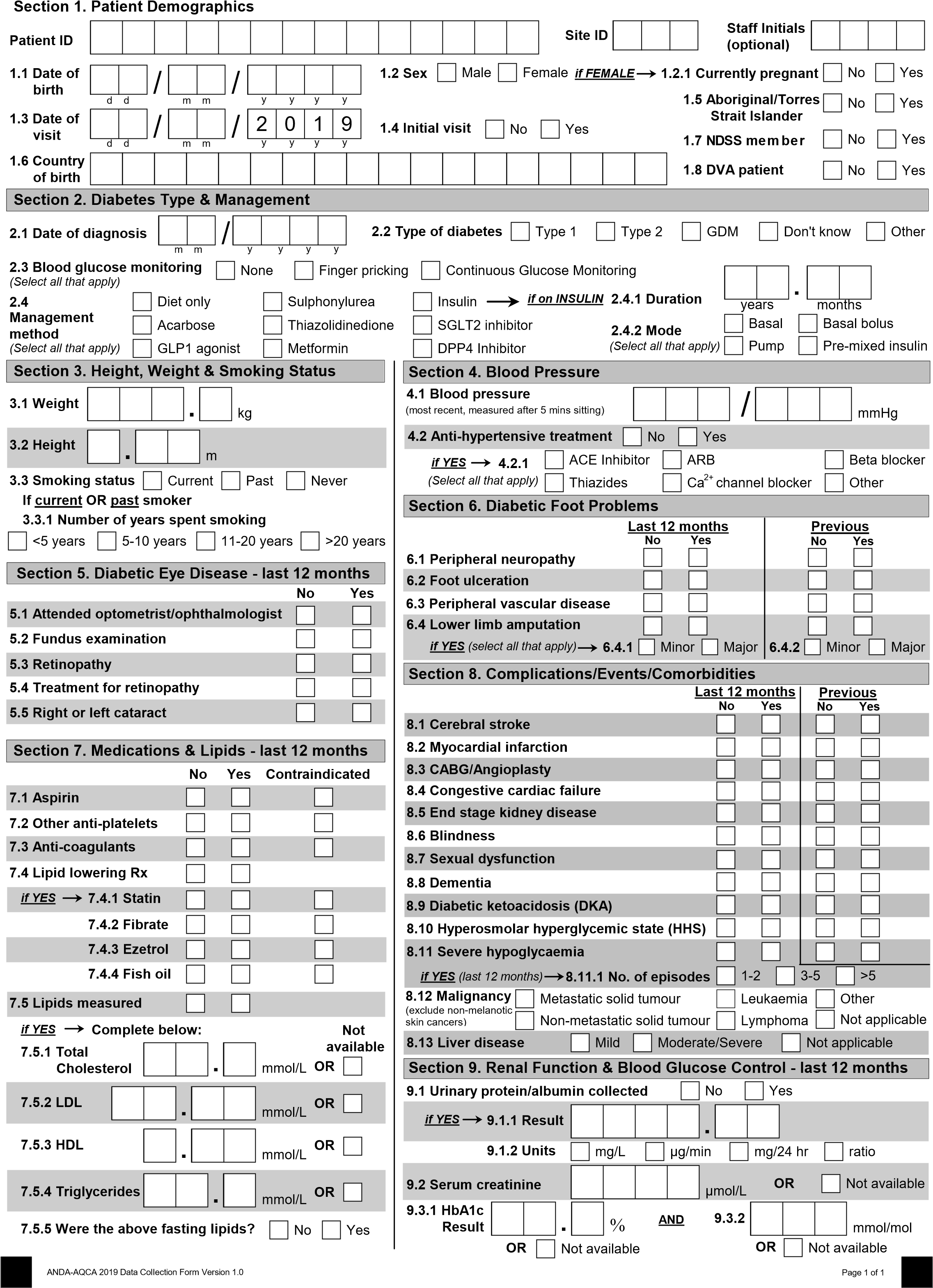

Supplement: S1 File — Supplied by Australian National Diabetes Audit (ANDA), Monash University, Melbourne, Australia. (DOCX) [file pone.0263511.s001.docx]
