# Supplementary material for: Exploring HbA1c variation between Australian diabetes centres: The impact of centre-level and patient-level factors
Source: PLoS One. 2022 Feb 4;17(2):e0263511. doi: 10.1371/journal.pone.0263511 (PMC8815864; doi:10.1371/journal.pone.0263511)
Supplement: S2 File — Sensitivity analysis, type 1 diabetes, excluding patients for whom the recorded visit was an initial visit. The relative contribution of centre-level factors and patient-level factors to HbA1c variation is shown for people with type 1 diabetes. 1COE: Centres of Excellence. 2eGFR: estimated Glomerular Filtration Rate (eGFR) (calculated according to the CKD-Epi formula detailed by Levey et al. [30]). 3 BMI: Body Mass Index (calculated in kg/m2 and categorised according to guidelines from the World Health Organization [31]). (DOCX) [file pone.0263511.s002.docx]

***S2.***

Table 5. Sensitivity analysis to exclude patients where data was collected at an initial visit, type 1 diabetes

| **Outcome variable: HbA1c percent** | **Coefficient** | **95% CI** | | **P** |
| --- | --- | --- | --- | --- |
| **Centre-level factors** |  |  |  |  |
| Centre type (ref: COE^1^ + tertiary) |  |  |  |  |
| Secondary care | -0.006 | -0.304 | 0.292 | 0.968 |
| Primary care | 0.478 | -0.081 | 1.037 | 0.094 |
| Site location (ref: metro) | 0.005 | -0.230 | 0.240 | 0.966 |
| Patient numbers (per 1 patient increase) | 0.002 | 0.000 | 0.003 | 0.036 |
| **Patient-level factors** |  |  |  |  |
| Diabetes duration (per 1-year increase) | -0.010 | -0.018 | -0.002 | 0.013 |
| eGFR^2^ (per 1 mL/min/1.73m2 increase) | 0.006 | 0.001 | 0.010 | 0.019 |
| Presence of diabetes complications (ref: no) | 0.521 | 0.298 | 0.745 | <0.001 |
| Hyperglycaemic episode (ref: no) | 0.543 | 0.337 | 0.748 | <0.001 |
| Smoking status (ref: current smoker) | -0.669 | -0.955 | -0.383 | <0.001 |
| Age category (ref: 18 - 39 years) |  |  |  |  |
| 40 - 59 years | 0.092 | -0.153 | 0.337 | 0.463 |
| 60 - 79 years | 0.048 | -0.271 | 0.366 | 0.768 |
| > 80 years | 0.399 | -0.424 | 1.222 | 0.342 |
| BMI^3^ category (ref: <18.49) |  |  |  |  |
| 18.5 - 24.99 | -0.463 | -1.220 | 0.294 | 0.231 |
| 25 - 29.99 | -0.658 | -1.415 | 0.100 | 0.089 |
| > 30 | -0.683 | -1.445 | 0.079 | 0.079 |
