# Supplementary material for: Exploring HbA1c variation between Australian diabetes centres: The impact of centre-level and patient-level factors
Source: PLoS One. 2022 Feb 4;17(2):e0263511. doi: 10.1371/journal.pone.0263511 (PMC8815864; doi:10.1371/journal.pone.0263511)
Supplement: S3 File — Sensitivity analysis, type 2 diabetes, excluding patients for whom the recorded visit was an initial visit. The relative contribution of centre-level factors and patient-level factors to HbA1c variation is shown for people with type 2 diabetes. 1COE: Centres of Excellence. 2 BMI: Body Mass Index (calculated in kg/m2 and categorised according to guidelines from the World Health Organization [31]). (DOCX) [file pone.0263511.s003.docx]

**S 3**

Table 6. Sensitivity analysis to exclude patients where data was collected at an initial visit, type 2 diabetes

| **Outcome variable: HbA1c percent** | **Coefficient** | **95% CI** | | **P** |
| --- | --- | --- | --- | --- |
| **Centre-level factors** |  |  |  |  |
| Centre type (ref: COE^1^ + tertiary) |  |  |  |  |
| Secondary care | 0.165 | -0.168 | 0.499 | 0.331 |
| Primary care | -0.449 | -0.763 | -0.135 | 0.005 |
| Site location (ref: metro) | -0.083 | -0.334 | 0.167 | 0.515 |
| Patient numbers (per 1 patient increase) | 0.001 | -0.001 | 0.004 | 0.200 |
| **Patient-level factors** |  |  |  |  |
| Total glucose lowering treatments (per 1 treatment increase) | 0.314 | 0.249 | 0.379 | <0.001 |
| Diabetes duration (per 1-year increase) | 0.015 | 0.008 | 0.022 | <0.001 |
| Hyperglycaemic episode (ref: no) | 0.705 | 0.337 | 1.073 | <0.001 |
| Smoking status (ref: current smoker) | -0.354 | -0.549 | -0.159 | <0.001 |
| Indigenous Australian status (ref: no) | 0.338 | 0.023 | 0.653 | 0.035 |
| Presence of diabetes complications (ref: no) | 0.155 | 0.028 | 0.282 | 0.017 |
| Age category (ref: 18 - 39 years) |  |  |  |  |
| 40 - 59 years | 0.207 | -0.075 | 0.490 | 0.150 |
| 60 - 79 years | -0.085 | -0.367 | 0.197 | 0.556 |
| > 80 years | -0.031 | -0.381 | 0.319 | 0.862 |
| BMI^2^ category (ref: <18.49) |  |  |  |  |
| 18.5 - 24.99 | 0.045 | -2.157 | 2.246 | 0.968 |
| 25 - 29.99 | 0.157 | -2.039 | 2.354 | 0.888 |
| > 30 | 0.256 | -1.938 | 2.451 | 0.819 |
