# Supplementary material for: Exploring HbA1c variation between Australian diabetes centres: The impact of centre-level and patient-level factors
Source: PLoS One. 2022 Feb 4;17(2):e0263511. doi: 10.1371/journal.pone.0263511 (PMC8815864; doi:10.1371/journal.pone.0263511)
Supplement: Attachment — Submitted filename: PLOS-Reviewer Comment-HbA1C-27Aug.docx [file pone.0263511.s004.docx]

In this cross sectional study authors analyzed a data of 1729 patients with type 1 diabetes and 4143 patients with type 2 diabetes collected through Australian National Diabetes Audit. The aim of this study was to identify the centre specific and patient specific factors associated with variation of hemoglobin A1C levels. Authors suggested interventions targeting patient level factors since patient level factors had higher impact on variability. Their finding was that centre location (Regional/Metropolitan) or centre type did not significantly contribute to the variation of hemoglobin A1C. However, in patients with type 1 diabetes, who are treated in centres with high volume patients had higher hemoglobin A1C. Patient level factors associated with higher HbA1C in type 1 diabetes included prior recorded hyperglycemic emergency episodes, presence of diabetes complications, and higher GFR. Patient level factors associated with lower hemoglobin A1C in type one diabetes included none smoking status, longer diabetes duration.

In patients with type 2 diabetes, being seen in primary care centre was associated with lower HbA1C. Patient volume or centre location did not significantly contribute to HbA1C. Factors associated with higher HbA1C in patients with type 2 diabetes included presence of diabetes complication, prior recorded hyperglycemia emergency, aboriginal and Torres Strait Islander status and number of glucose lowering agents. The only patient level factor associated with lower hemoglobin A1C in type 2 diabetes was non-smoking status.

Author brought up the important concept to identify patient specific factors to target delivery of care. However, clarification is needed for their data and interpretation of their findings.

1) **Effect of centre-level factors on HbA1c variation:** Authors have identified that one reason might be that patient with stable type 2 diabetes are managed in primary care and more difficult cases are being referred to more specialized secondary or tertiary care centre. Authors have also cautioned about their finding in people with type 1 diabetes that patient volume association with her hemoglobin A1C was because tertiary care centres are located in more densely populated areas, where they see clinically complex patients.

There is no clear description of what is the available service at present to support authors’ interpretation. There is no analysis or data on what kind of care is delivered in these centres according to Chronic Care Model such as self-management support, clinical information system, or decision support. Another important information that will strengthen the paper is the frequency of contact with the health care providers, as well as ratio of patients and providers rather than volume of patients only. This information might also give the answer why diabetes centre location was not associated with variation in HbA1c and authors finding is different than other study. (Page 16-Line249)

**2) Effect of patient-level factors on HbA1c variation**

a) **Hyperglycaemic emergencies and HbA1c variation**: This paragraph is like a textbook. This needs revision to make relevant and explain authors finding.
b) **The presence of diabetes complications and HbA1c variation:** “The presence of diabetes complications was associated with higher HbA1c levels irrespective of diabetes type.” The logical explanation is higher HbA1C is associated with diabetes complication. It is important to identify what is the cause and what is the effect to appropriately plan care delivery.
c) **Smoking status and Hba1c variation:** Authors found that being a non-smoker was associated with reductions in HbA1c. People without diabetes in ANDA cohort also showed similar result. Authors finding sure emphasize importance of smoking cessation for everyone.
d) **Aboriginal and Torres Strait Islander status and HbA1c variation**; Since there is higher prevalence of type 2 diabetes in Aboriginal and Torres Strait Islander status, authors very appropriately suggested implementation of codeveloped resources and culturally and linguistically appropriate diabetes education programs. It will be important to know what is the current state of therapeutic relationship between the centers and these groups of patients. Is there any purposeful process of learning offered to the health-care workers to integrate specific contexts who are involved in care of these patients?
e) **Glucose-lowering medications and HbA1c variation**: It is not clear if insulin is included in the medications. Authors interpretation is “this is reflective of clinical practice where despite intensification of treatment and multiple pharmacological agents, some patients do not see clinically significant declines in HbA1c” (Page 20-line 330). Really? Could it be due to well-known providers’ “therapeutic inertia” - failure to initiate or intensify therapy in a timely manner resulting uncontrolled hyperglycemia in patients with type 2 diabetes. At times multiple agents are added just to defer insulin initiation.
